# Delayed bowel perforation following suprapubic catheter insertion

**DOI:** 10.1186/1471-2490-4-16

**Published:** 2004-12-15

**Authors:** Shwan J Ahmed, Ajay Mehta, Peter Rimington

**Affiliations:** 1 Department of Urology, Royal Surrey County Hospital, Egerton Road, Guildford, UK; 2 Department of Urology, Eastbourne District General Hospital, Kings Way, Eastbourne, UK

## Abstract

**Background:**

Complications of suprapubic catheter insertion are rare but can be significant. We describe an unusual complication of a delayed bowel perforation following suprapubic catheter insertion.

**Case presentation:**

A gentleman presented with features of peritonitis and feculent discharge along a suprapubic catheter two months after insertion of the catheter.

**Conclusion:**

Bowel perforation is the most feared complication of suprapubic catheter insertion especially in patients with lower abdominal scar. The risk may be reduced with the use of ultrasound scan guidance.

## Background

Suprapubic catheterization is a common urological procedure. Complications of catheter insertion are uncommon but can be serious including bowel perforation or obstruction. We describe an unusual complication of delayed bowel perforation after suprapubic catheter insertion.

## Case presentation

An 86 year old gentleman had a suprapubic catheter inserted for bladder outlet obstruction. This was done under a local anaesthetic using the standard Lawrence Add-a-cath^® ^trocar with ultra sound guidance to measure the bladder volume which was estimated as 500 ml. He had been diagnosed with poorly differentiated carcinoma of the prostate six years earlier and had undergone radical radiotherapy, bilateral sub-capsular orchidectomy and transurethral resection of prostate gland during the interim period. He had bilateral ureteric stents inserted for obstructive uropathy six months earlier. He had a past history of abdomino-perineal resection for rectal carcinoma fourteen years earlier as a curative procedure.

**Figure 1:**
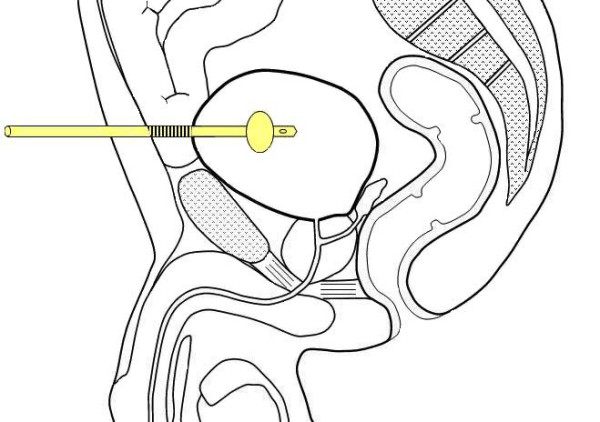
Suprapubic catheter traverse through a small bowel loop between the bladder and the abdominal wall.

He returned for the first change of supra pubic catheter to the Urology suite in two months time. The catheter was changed easily by the specialist nurse and the patient was discharged home. He returned about ten hours later with features of peritonitis and feculent discharge along the supra pubic catheter. He underwent an emergency explorative laparotomy. A loop of small bowel – adhered to the scar – was placed between the anterior abdominal wall and the bladder. The supra pubic tract was seen to pass through and penetrate the loop in two places before going into the urinary bladder [figure 1]. There was excessive fibrosis of the bowel segment in the area surrounding the perforation sites. Resection of the affected bowel segment and end-to-end anastomosis was undertaken. An indwelling urethral catheter was left in situ. He made a complete recovery and has been left with the urethral catheter.

## Conclusions

Perforation of the abdominal viscera is well documented as a rare but important major complication of suprapubic cystostomy [1,2]. To our knowledge, only one case of delayed bowel perforation has been reported [3] three months after the actual catheter insertion. The likely mechanism is the injury occurred during the original insertion. The catheter and the ensuing inflammatory fibrosis sealed the perforation. On removal of the catheter during the change, the sealed perforation opened up.

Our case explains the increased risk of bowel damage during suprapubic catheterization in patients with history of previous lower abdominal surgery as the bowel frequently adheres to the scar. In one study, it was found that 59% of patients with midline laparotomy incision have anterior abdominal wall adhesions [4]. Therefore, patients with lower abdominal scar should only have suprapubic catheter placement under ideal conditions to reduce risk of bowel perforation. Patients must have adequately distended bladder and placed in Trendelenburg position. We do recommend that the procedure to be performed by a skilled operator using ultrasound scan to look for bowel loops between the bladder and anterior abdominal wall. If bowel loops are present or if ultrasound facilities are not available, then open cystostomy method should be considered. The first change of the catheter should be done in the urology department rather than in the community. Patients returning after having their first catheter change with features of localised peritonitis (lower abdominal pain, high temperature and raised White Blood Cell count) should alert the urologist for the possibility of bowel perforation.

## Authors' competing interests

The author(s) declare that they have no competing interests.

## Author's contributions

SA Collected the data; drafted and revised the manuscript and drew the illustration. AM helped to draft and revise the manuscript and PR was the main surgeon and helped to revise the manuscript.

## Pre-publication history

The pre-publication history for this paper can be accessed here:
